# Phallusiasterol C, A New Disulfated Steroid from the Mediterranean Tunicate *Phallusia fumigata*

**DOI:** 10.3390/md14060117

**Published:** 2016-06-18

**Authors:** Concetta Imperatore, Maria Senese, Anna Aiello, Paolo Luciano, Stefano Fiorucci, Claudio D’Amore, Adriana Carino, Marialuisa Menna

**Affiliations:** 1 The NeaNat Group, Department of Pharmacy, University of Naples “Federico II”, Via D. Montesano 49, Napoli 80131, Italy; cimperat@unina.it (C.I.); maria.senese@unina.it (M.S.); aiello@unina.it (A.A.); pluciano@unina.it (P.L.); 2 Department of Surgical and Biomedical Sciences, Faculty of Medicine, University of Perugia, Via Gambuli 1, S. Andrea delle Fratte, Perugia 06132, Italy; fiorucci@unipg.it (S.F.); claudiodamore1983@gmail.com (C.D.); adriana.carino@hotmail.it (A.C.)

**Keywords:** sulfated sterol, tunicates, *Phallusia fumigata*, structure elucidation, nuclear receptors, PXR modulator

## Abstract

A new sulfated sterol, phallusiasterol C (**1**), has been isolated from the Mediterranean ascidian *Phallusia fumigata* and its structure has been determined on the basis of extensive spectroscopic (mainly 2D NMR) analysis. The possible role in regulating the pregnane X receptor (PXR) activity of phallusiasterol C has been investigated; although the new sterol resulted inactive, this study adds more items to the knowledge of the structure-PXR regulating activity relationships in the case of sulfated steroids.

## 1. Introduction

**Figure 1:**
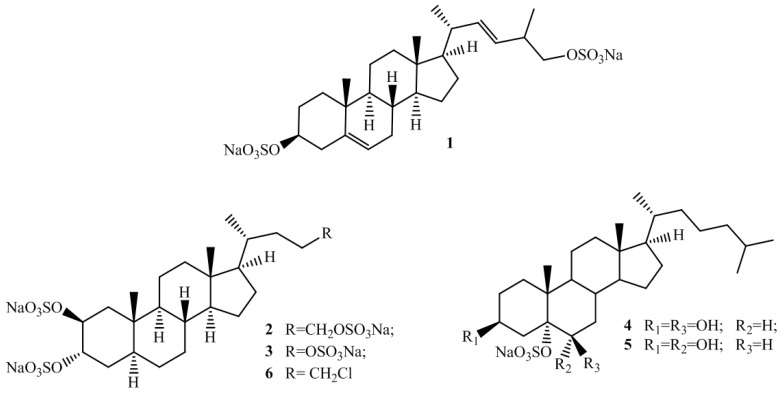
Structures of phallusiasterol C (**1**); solomonsterols A (**2**) and B (**3**); phallusiasterols A (**4**) and B (**5**); and chalinulasterol (**6**).

Marine steroids display an extraordinary chemical diversity, mainly resulting from extensive oxygenation of the basic carbon skeleton, cleavage and/or re-arrangement on the rings of tetracyclic nucleus, and side chain alteration [1,2,3,4,5]. In fact, “usual” sterols usually have a 3-hydroxycholestane or 3-hydroxy-Δ^5^-cholestane nuclei and a C8–C10 side chain [6], whereas marine sterols have been isolated featuring either or both of the distinctive features of (i) carbon side chains in the C0 to C12 range, involving loss of carbon atoms or their addition at positions other than C-24, and (ii) multiple oxygenation of the side chain and/or the nucleus [7]. More than 1600 new steroidal structures have been isolated from sea organisms, mainly from marine invertebrates, including algae, porifera, and tunicates [6,7]. The structural diversity of these metabolites, chiefly the polar sterols, is reflected in a diverse array of different pharmacological properties, including cytotoxic [8], spermatostatic [9], antifeedant [10], anti-inflammatory [11], and anti-human cytomegalovirus (HCMV) [12] activities; particularly, the role of marine sponge steroids as nuclear receptor ligands has been recently highlighted [13]. Sulfated steroids endowed with dual farnesoid X receptor (FXR) and pregnane X receptor agonism–antagonism have been identified, like solomonsterols A (**2**) and B (**3**), isolated from the sponge *Theonella swinhoei* [14,15], and phallusiasterol A (**4**) that we have recently isolated from the Mediterranean ascidian *Phallusia fumigata*, together with its C-6 epimer phallusiasterol B (**5**) (Figure 1) [16]. In particular, Investigation of the effects of phallusiasterols on the activity of pregnane-X-receptor (PXR) revealed that phallusiasterol A induces PXR transactivation in HepG2 cells and stimulates the expression of the PXR target genes CYP3A4 and MDR1 in the same cell line whereas phallusiasterol B was inactive. This study confirmed the role of steroids in regulating the nuclear receptors (NR) activity, and evidenced a crucial reliance on some structural features, like the configuration at C-6, of the ligand-receptor binding.

Re-investigation of a new collection of *P. fumigata* led to the isolation, from the more polar fraction of the butanol extract, of a new disulfated sterol, named phallusiasterol C (**1**, Figure 1), of which the structure was elucidated by spectroscopic means (see Figures S1–S6 in Supplementary Materials). The possible role in regulating the PXR activity of phallusiasterol C has been investigated, too. Despite the structural similarity with solomonsterol A for the short and sulfated side chain, compound **1** was inactive as PXR agonist, revealing important structural requirements for the PXR nuclear receptor activity of sulfated steroidal structures.

## 2. Results and Discussion

### 2.1. Isolation and Structure Elucidation

The negative ion HR ESI mass spectrum of **1** displayed a [M − Na^+^]^−^ pseudo-molecular ion peak at *m/z* 567.2054 (see Figure S7 in Supplementary Materials); the formula C_26_H_40_NaO_8_S_2_^−^ was thus deduced for this ion (calcd. 567.2057), which indicated six unsaturation degrees. The ESI and MS/MS fragmentation pattern of **1** revealed the presence of two sulfate groups from the peaks 545 (M^−^ in hydrogen form), 272 (double charged species), and 447 [M − NaHSO_4_ − Na^+^]^−^.

The ^1^H NMR spectrum (CD_3_OD) of **1** suggested its steroidal structure, with two up-field methyl singlets at δ 0.72 (H_3_-18) and 1.03 (H_3_-19) and one methyl doublet at δ 1.03 (*J* = 6.4 Hz, H_3_-21). Other proton resonances, combined with ^13^C NMR and mass data, evidenced the presence of one secondary (δ_H_ 4.14, dddd, *J* = 11.4, 11.4, 4.8, 4.8 Hz, δ_C_ 80.0, CH) and one primary (δ_H_ 3.88, dd, *J* = 9.3, 6.0 Hz; δ_H_ 3.74, dd, *J* = 9.3, 7.8 Hz, δ_C_ 73.8, CH_2_) sulfoxy groups in the molecule. Furthermore, three olefin signals resonating in the proton spectrum at δ 5.38 (dd, *J* = 6.9, 3.3 Hz, 1H, H-6), 5.35 (dd, *J* = 15.3, 8.5 Hz, 1H, H-22), and 5.28 (dd, *J* = 15.3, 7.0 Hz, 1H, H-23) were assigned to two double bonds, one trisubstituted and one disubstituted, based on the four down-field resonances present in the ^13^C NMR spectrum at δ_C_ 141.5 (C), 123.4 (CH), 138.5 (CH) and 130.2 (CH). Interpretation of COSY, HSQC and HMBC 2D NMR experiments allowed the steroidal backbone to be assembled and all the protons and carbons of the tetracyclic system to be assigned to the relevant resonances (see Table 1 and Figures S3–S5 in Supplementary Materials).

The location of the secondary sulfoxy group at C-3 and the Δ^5(6)^ position of the endocyclic double bond were thus deduced. The HMBC correlation peaks of the methyl protons H_3_-19 with C-1, C-5, C-9, and C-10 and of H_3_-18 with C-12, C-13, C-14, and C-17 located the A/B and C/D ring junctions and completed the planar structure determination of the steroid ring system.

**Figure 2:**
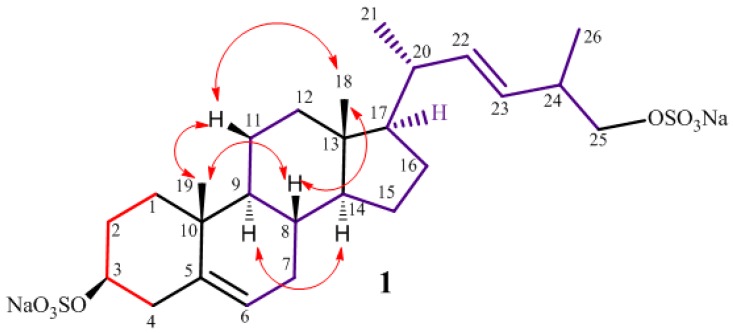
COSY segments (represented as colored bonds) and key ROESY correlations (arrows) for the phallusiasterol C (**1**).

The nature of the side chain was easily deduced by analysis of COSY map. A single ^1^H-^1^H spin system was delineated, starting at the methyl doublet at δ 1.03 (H_3_-21) which was correlated to the proton at δ 2.06 (H-20). The latter was coupled with both the H-17 methine proton (δ 1.17) and the olefinic proton at δ 5.35 (H-22), in turn coupled with the other olefin proton at δ 5.28 (H-23). This indicated the Δ^22^ position of the remaining double bond, of which the *E*-configuration was suggested by the *J* value (15.3 Hz) of H-22 and H-23. The coupling of H-23 to the multiplet at δ 2.44 (H-24), which in turn was coupled both to the methyl protons at δ 1.02 (H_3_-26) and the sulfoxy methylene protons at δ 3.88 (H-25a) and 3.74 (H-25b), completed the assignment, indicating a 24-methyl-25-sulfoxy C26 side chain for **1** (Figure 2).

The relative stereochemistry of phallusiasterol C (**1**) with the B/C and C/D *trans* ring junctions, was established through analysis of ROESY data and coupling constant analysis. The axial orientation of H-8, H-9 and H-14 was apparent from their respective coupling constants that of both the angular methyl groups from their ROESY correlations with H-8 and the axial H-11β (see Figure S6 in Supplementary Materials). On this skeleton, the 3β-sulfoxy configuration was assigned on the basis of the coupling pattern of H-3 (dddd) indicating its axial orientation. The orientation of substituents at C-17 and C-20 in phallusiasterol C (**1**) was presumed to be the same as in related polyhydroxysterols due to very similar values of carbon chemical shifts around these carbon atoms [17,18,19]. According to this information, the structure of phallusiasterol C (**1**) was established as (22*E*)-26,27-dinor-24ξ-methyl-cholesta-5,22-dyen-3β,25-diyl-3,25-sodium disulfate.

### 2.2. Biological Evaluation

**Figure 3:**
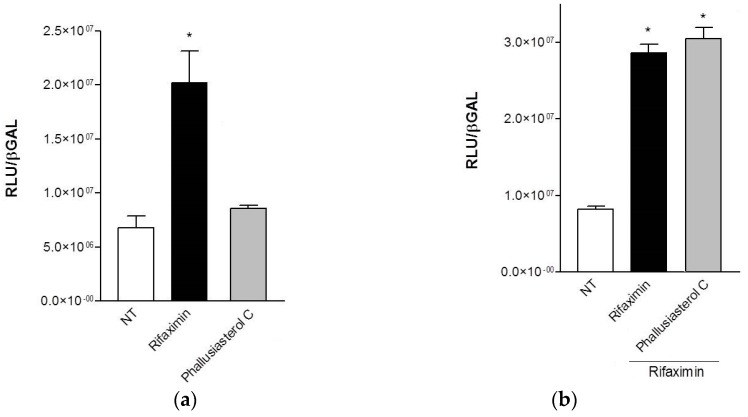
(**a**, **b**) Luciferase reporter assay. HepG2 cells were transiently transfected with pSG5-PXR, pSG5-RXR, pCMV-βgalactosidase and p(CYP3A4)-TK-Luc vectors and then stimulated with (**a**) 10 μM rifaximin or phallusiasterol C (**1**) for 18 h, or (**b**) 10 μM rifaximin alone or in combination with 50 μM of compound **1**. Relative Luciferase Units were normalized with β-galactosidase Units (RLU/βgal). All experiments were performed in triplicate. NT, not treated cells. R, Rifaximin. * *p* < 0.05 *versus* NT cells. Data are mean ± SE.

The possible role in regulating the PXR activity of phallusiasterol C has been investigated. A transactivation assay on HepG2 cells, a human hepatocarcinoma cell line, has been performed, as described in the Experimental Section. Despite the structural similarity with solomonsterol A for the short and sulfated side chain, compound **1** was inactive as PXR agonist. In addition, it also failed to reverse the induction of luciferase activity caused by rifaximin, indicating that it was not a PXR antagonist (Figure 3). Similar results have been obtained by analyzing the effect exerted by **1** in terms of regulation of PXR mediated induction of two PXR target genes, CYP3A4 and MDR1, in the same cell line; in this assay, compound **1** failed to induce the expression of both target genes (data not shown).

These results, although negative, add more items to the knowledge of the structure-PXR regulating activity relationship in the case of sulfated steroids. In the binding model proposed for solomonsterol A to the PXR receptor, a clear stabilizing interaction of the side chain sulfate group with the positively charged Lys210 has been observed [13]. This model was supported by studies on chalinulasterol, which has a close structural relationship with solomonsterol A and differs from the latter compound in having a chlorine atom instead of a sulfate function at position C-24 of the side chain [20]. Chalinulasterol lacked any PXR modulating activity and, thus, an essential role of the sulfate group present in the side chain has been proposed. However, the activity of phallusiasterol A (**4**) as PXR agonist, comparable to that of rifaximin, a well characterized ligand for the human PXR, contradict this assumption since it features a “regular” sterol side chain (Figure 1). Instead, the feature and/or the shape of the region around the A/B ring junction seems to be critical; in fact, both phallusiasterol B (**5**), which differs from phallusiasterol A only for the configuration at C-6, and phallusiasterol C, featuring the Δ^5(6)^ double bond, lacked any PXR modulating activity (Figure 1).

## 3. Materials and Methods

### 3.1. General Experimental Procedures

High-resolution ESI-MS analyses were performed on a Thermo LTQ Orbitrap XL mass spectrometer (Thermo-Fisher, San Josè, CA, USA). The spectra were recorded by infusion into the ESI (Thermo-Fisher) source using MeOH as solvent. Optical rotations were measured at 589 nm on a Jasco P-2000 polarimeter (Jasco, Inc., Easton, MD, USA) sing a 10-cm microcell. ^1^H (700 MHz) and ^13^C (175 MHz) NMR spectra were recorded on a Agilent INOVA spectrometer (Agilent Technology, Cernusco sul Naviglio, Italy) equipped with a ^13^C enhanced HCN Cold Probe; chemical shifts were referenced to the residual solvent signal (CHD_2_OD: δ_H_ = 3.31, δ_C_ = 49.0). For an accurate measurement of the coupling constants, the one-dimensional ^1^H NMR spectra were transformed at 64 K points (digital resolution: 0.09 Hz). Homonuclear ^1^H connectivities were determined by COSY experiment. Through-space ^1^H connectivities were evidenced using a ROESY experiment with a mixing time of 500 ms. Two and three bond ^1^H-^13^C connectivities were determined by gradient 2D HMBC experiments optimized for a ^2,3^*J* of 8 Hz. ^3^*J*_H-H_ values were extracted from 1D ^1^H NMR. Medium-pressure liquid chromatographies (MPLC) were performed on a Büchi 861 apparatus (Buchi Italia s.r.l., Cornaredo, Italy) with octadecyl-functionalized silica gel (200–400 mesh) packed column. High performance liquid chromatography (HPLC) separations were achieved on a Shimadzu LC-10AT (Shimadzu, Milan, Italy) apparatus equipped with a Knauer K-2301 (LabService Analytica s.r.l., Anzola dell’Emilia, Italy) refractive index detector.

### 3.2. Collection, Extraction, and Isolation

Specimens of *Phallusia fumigata* were collected in May 2010 in the bay of Pozzuoli (Napoli, Italy). The samples were frozen immediately after collection and stored at −20 °C until extraction. A reference specimen is deposited at the Department of Pharmacy, University of Naples. The fresh thawed animals (402 g of dry weight after extraction) were homogenized and extracted twice with methanol and then twice with chloroform (4 × 200 mL). The combined extracts were concentrated in vacuo, and the resulting aqueous residue was extracted with EtOAc and subsequently with *n*-BuOH. Separation of the *n*-BuOH soluble material (3.06 g) was achieved by gradient RP-18 silica gel MPLC (Sigma-Aldrich, Inc., St. Louis, MO, USA) (H_2_O → MeOH → CHCl_3_) to yield nine fractions A–I. The fraction eluted with H_2_O/MeOH 3:7 *v*/*v*, (fraction D, 98.2 mg) was chromatographed by HPLC on a RP-18 column (polar-RP 5 m, 250 × 4.60 mm) eluting with H_2_O/MeOH 35:65 (*v*/*v*), yielding a fraction mainly composed of **1** (5.6 mg) which has been further purified by HPLC on a RP-18 column (Synergy 4 m, 250 × 4.60 mm), eluting with H_2_O/MeOH 3:7, thus affording phallusiasterol C (1.1 mg) as pure compound.

### 3.3. Phallusiasterol C (**1**)

Colorless amorphous solid, ${\left[\alpha \right]}_{D}^{25}$ −4.0 (*c* 0.1, CH_3_OH); HRESIMS (negative ion mode, CH_3_OH) *m/z* 567.2054 ([M − Na]^−^, calcd. for C_26_H_40_NaO_8_S_2_^−^ 567.2057); ^1^H and ^13^C NMR (CD_3_OD): see Table 1.

### 3.4. Transactivation Experiments

To investigate the PXR mediated transactivation, HepG2 cells were plated in a 24-wells plate, at 5 × 10^4^ cells/well, and transiently transfected with 75 ng of pSG5-PXR, 75 ng of pSG5-RXR, 125 ng of pCMV-β-galactosidase, and with 250 ng of the reporter vector pCYP3A4promoter-TKLuc, using Fugene HD transfection reagent (Roche). At 24 h post-transfection, cells were primed 18 h with Rifaximin and **1** (10 μM) or with the combination of Rifaximin (10 μM) plus compound **1** (50 μM). After treatments, cells were lysed in 100 μL Lysis Buffer (25 mM TRIS-phosphate pH 7.8; 2 mM DTT; 10% glycerol; 1% Triton X-100) and 20 μL cellular lysate was assayed for Luciferase activity using the Luciferase Assay System (Promega Corporation, Madison, WI, USA). Luminescence was measured using Glomax 20/20 automated luminometer (Promega Corporation). Luciferase activities were normalized for transfection efficiencies by dividing the Luciferase relative light units (RLU) by β-galactosidase activity (βgal) expressed from cells co-transfected with pCMVβgal. All experiments were performed in triplicate.

### 3.5. Cells Culture, RNA Extraction and Real-Time PCR

HepG2 cells were cultured at 37 °C in E-MEM supplemented with 10% FBS, 1% l-glutamine and 1% penicillin/streptomycin. To evaluate PXR target genes expression, serum starved HepG2 cells were stimulated for 18 h with Rifaximin and compound **1** (10 μM). Total RNA was extracted using the TRIzol reagent (Invitrogen, Life Technology, Carlsband, CA, USA), purified of the genomic DNA by DNAase I treatment (Invitrogen, Life Technology) and random reverse-transcribed with Superscript II (Invitrogen, Life Technology). A 10 ng template was amplified using the following reagents: 0.2 μM of each primer and 10 μL of KAPA SYBR FAST Universal qPCR Kit (KAPA BIOSYSTEMS, Woburn, MA, USA). All reactions were performed in triplicate and the thermal cycling conditions were: 3 min at 95 °C, followed by 40 cycles of 95 °C for 15 s, 58 °C for 20 s and 72 °C for 30 s. The relative mRNA expression was calculated and expressed as 2^−(ΔΔ*Ct*)^. Primers used for qRT-PCR were: hGAPDH: GAAGGTGAAGGTCGGAGT and CATGGGTGGAATCATATTGGAA; hCYP3A4: CAAGACCCCTTTGTGGAAAA and CGAGGCGACTTTCTTTCATC; hMDR1: GTGGGGCAAGTCAGTTCATT and CTTCACCTCCAGGCTCAGT.

### 3.6. Statistical Analysis

All values are expressed as means ± standard error (SE) of n observations/group. Comparisons of two groups were made with a one-way ANOVA with *post hoc* Tukey’s test. Differences were considered statistically significant at values of *p* < 0.05.

## 4. Conclusions

The structure of phallusiasterol C (**1**), a new disulfated steroid isolated from the Mediterranean tunicate *Phallusia fumigata* was elucidated using mass spectrometry and NMR experiments. Phallusiasterol C (**1**) is the first example of a sterol with a sulfated (22*E*)-26,27-dinor-24-methyl-25-hydroxy side chain from tunicates; up to now, only one sterol with the same side chain has been isolated from the starfish *Ctenodiscus crispatus* [21] and only few similar polyhydroxylated steroids containing an analogous, but sulfate-free, side chain from the starfishes and from sea gorgonian have been characterized [22,23,24,25,26]. However, the occurrence of sterols with side chains of the 24-methyl-27-nor- and 24-methyl-26,27-dinorcholestane types in nearly every marine invertebrate phylum is reported and this suggests that these sterols could be of a dietary origin. Moreover, it has been reported that marine C26 sterols (*i.e.*, 26,27-dinor-24-methylcholestane) originate from phytoplankton [6,7,25,27,28]. Therefore, the occurrence of (**1**) in *P. fumigata* may be of ecological interest, since it could be an indicator of the tunicate’s ability to oxidize dietary sterols.

Investigation of the possible role of phallusiasterol C as modulator of the PXR nuclear receptor revealed important structural requirements for the PXR nuclear receptor activity of sulfated steroidal structures.

## Figures and Tables

**Table 1:** ^1^H (700 MHz) and ^13^C (125 MHz) NMR data for phallusiasterol C (**1**) in CD_3_OD.

Position	δ_H_ (mult., *J* in Hz)	δ_C_	HMBC	Pos.	δ_H_ (mult., *J* in Hz)	δ_C_	HMBC
**1β**	1.90, dt, (13.5, 3.6)	38.4	2, 5, 10, 19	**-**	-	-	-
**1α**	1.10, m		2, 3, 10, 19	**13**	-	43.4	-
**2β**	1.63, m ^a^	29.9	1, 3, 10	**14α**	1.02 ^b^	58.2	8, 13, 15, 16, 18
**2α**	2.07, m ^a^		1, 3, 10	**15β**	1.07, m ^a^	25.3	13, 14, 16, 17
**3α**	4.14, dddd, (11.4, 11.4, 4.8, 4.8)	80.0	1, 2, 4	**15α**	1.58 ^a,b^		8, 14, 16
**4β**	2.34, dd, (13.2, 11.4)	40.3	2, 3, 5, 6, 10	**16β**	1.28, m ^a^	29.7	13, 15, 17
**4α**	2.53, ddd, (13.2, 4.8, 2.2)		2, 3, 5	**16α**	1.71, m ^a^		13, 17, 20
**5**	-	141.5	-	**17**	1.17, m	57.2	13, 15, 16, 20, 22
**6**	5.38, dd, (6.9, 3.3)	123.4	4, 7, 8, 10	**18**	0.72, s	12.5	12, 13, 14, 17
**7β**	1.98, dt, (13.4, 6.9, 3.3)	33.0	5, 6, 8, 9	**19**	1.03, s	19.7	1, 5, 9, 10
**7α**	1.55 ^b^		5, 8, 14	**20**	2.06, m	41.5	17, 21, 22, 23
**8β**	1.48, m (qd, 10.7, 4.3)	33.2	7, 9, 14	**21**	1.03, d, (6.4)	19.7	17, 20, 22
**9α**	0.96, ddd (13.2, 10.7, 4.3)	51.7	8, 10, 11, 19	**22**	5.35, dd, (15.3, 8.5)	138.5	20, 21, 23, 24
**10**	-	37.7	-	**23**	5.28, dd, (15.3, 7.0)	130.2	20, 21, 22, 25, 26
**11β**	1.55 ^b^	22.1	9, 10, 12	**24**	2.44, m	37.6	22, 23, 25, 26
**11α**	1.51, m		9, 10, 12	**25a**	3.88, dd, (9.3, 6.0)	73.8	23, 24, 26
**12β**	2.01, dt, (12.9, 3.5)	41.0	11, 14	**25b**	3.74, dd, (9.3, 7.8)		23, 24, 26
**12α**	1.18, m		9, 13, 14	**26**	1.02, d, (6.2)	21.2	23, 24, 25

^a^ Assignments may be interchanged; ^b^ Overlapped by other signals.

## Supplementary Materials

The following are available online at www.mdpi.com/1660-3397/14/6/117/s1, Figure S1: ^1^H-NMR spectrum of phallusiasterol C (**1**) in CD_3_OD, Figure S2: ^13^C-NMR spectrum of phallusiasterol C (**1**) in CD_3_OD, Figure S3: COSY spectrum of phallusiasterol C (**1**) in CD_3_OD, Figure S4: HSQC spectrum of phallusiasterol C (**1**) in CD_3_OD, Figure S5: HMBC spectrum of phallusiasterol C (**1**) in CD_3_OD, Figure S6: ROESY spectrum of phallusiasterol C (**1**) in CD_3_OD, Figure S7: Negative-ion HRESI mass spectrum of phallusiasterol C (**1**).

## Abbreviations

The following abbreviations are used in this manuscript:

PXR	pregnane-X-receptor
HCMV	human cytomegalovirus
FXR	farnesoid X receptor
HepG2	hepatoma G2
HR ESI	High-resolution electrospray ionisation
COSY	Two dimensional ^1^H correlation
HSQC	^1^H-detected heteronuclear single-quantum coherence
HMBC	^1^H-detected heteronuclear multiple-bond correlation
ROESY	Rotating-frame Overhauser Spectroscopy

## References

[1] Lakshmi V., Kumar R. (2009). Metabolites from Sinularia species. Nat. Prod. Res..

[2] Sarma N.S., Krishna M.S., Pasha S.G., Rao T.S.P., Venkateswarlu Y., Parameswaran P.S. (2009). Marine Metabolites: The Sterols of Soft Coral. Chem. Rev..

[3] Zhang W., Guo Y.W., Gu Y.C. (2006). Secondary metabolites from the South China Sea invertebrates: Chemistry and biological activity. Curr. Med. Chem..

[4] Sica D., Musumeci D. (2004). Secosteroids of marine origin. Steroids.

[5] Sun P., Meng L.Y., Tang H., Liu B.S., Li L., Yi Y., Zhang W. (2012). Sinularosides A and B, Bioactive 9,11-Secosteroidal Glycosides from the South China Sea Soft Coral *Sinularia humilis* Ofwegen. J. Nat. Prod..

[6] Goad L.J., Scheuer P.J. (1978). The sterols of marine invertebrates: Composition, biosynthesis and metabolites. Marine Natural Products, Chemical and Biological Perspectives.

[7] Schmitz F.J., Scheuer P.J. (1978). Uncommon marine steroids. Marine Natural Products, Chemical and Biological Perspectives.

[8] Yan X.H., Lin L.P., Ding J., Guo Y.W. (2007). Methyl spongoate, a cytotoxic steroid from the Sanya soft coral *Spongodes* sp.. Bioorg. Med. Chem. Lett..

[9] Tillekeratne L.M.V., Liyanage G.K., Ratnasooriya W.D., Ksebati M.B., Schmitz F.J. (1989). A new spermatostatic glycoside from the soft coral *Sinularia crispa*. J. Nat. Prod..

[10] Li R., Shao C.L., Qi X., Li X.B., Li J., Sun L.L., Wang C.Y. (2012). Polyoxygenated sterols from the South China Sea soft coral *Sinularia* sp.. Mar. Drugs.

[11] Cheng S.Y., Huang Y.C., Wen Z.H., Hsu C.H., Wang S.K., Dai C.F., Duh C.Y. (2009). New 19-oxygenated and 4-methylated steroids from the Formosan soft coral *Nephthea chabroli*. Steroids.

[12] Chen W.H., Wang S.K., Duh C.Y. (2011). Polyhydroxylated Steroids from the Bamboo Coral *Isis hippuris*. Mar. Drugs.

[13] Blumberg B., Sabbagh W., Juguilon H., Bolado J., van Meter C.M., Ong E.S., Evans R.M. (1998). SXR, a novel steroid and xenobiotic-sensing nuclear receptor. Genes Dev..

[14] Festa C., de Marino S., D’Auria M.V., Bifulco G., Renga B., Fiorucci S., Petek S., Zampella A. (2011). Solomonsterols A and B from *Theonella swinhoei*. The first example of C-24 and C-23 sulfated sterols from a marine source endowed with a PXR agonistic activity. J. Med. Chem..

[15] Mencarelli A., D’Amore C., Renga B., Cipriani S., Carino A., Sepe V., Perissutti E., D’Auria M.V., Zampella A., Distrutti E. (2013). Solomonsterol A, a marine pregnane-X-receptor agonist, attenuates inflammation and immune dysfunction in a mouse model of arthritis. Mar. Drugs.

[16] Imperatore C., D’Aniello F., Aiello A., Menna M., Fiorucci S., D’Amore C., Sepe V. (2014). Phallusiasterols A and B: Two new sulfated sterols from the Mediterranean tunicate *Phallusia fumigata* and their effects as modulators of the PXR receptor. Mar. Drugs.

[17] Aiello A., Fattorusso E., Menna M., Carnuccio R., Iuvone T. (1995). New cytotoxic steroids from the marine sponge *Dysidea fragilis* coming from the lagoon of Venice. Steroids.

[18] Fujimoto Y., Yamada T., Ikekawa N. (1985). Pyridine-induced deshielding of 4-methylene protons for the determination of C-6 stereochemistry of sterols having a 5α,6-diol moiety. Revision of the C-6 stereochemistry of marine sterol isolated from a sponge, *Dysidea* sp.. Chem. Pharm. Bull. (Tokyo).

[19] Notaro G., Piccialli V., Sica D., Corriero G. (1991). 3β,5α,6β-Trihydroxylated sterols with a saturated nucleus from two populations of the marine sponge Cliona copiosa. J. Nat. Prod..

[20] Teta R., Della Sala G., Renga B., Mangoni A., Fiorucci S., Costantino V. (2012). Chalinulasterol, a Chlorinated Steroid Disulfate from the Caribbean Sponge *Chalinula molitba*. Evaluation of Its Role as PXR Receptor Modulator. Mar. Drugs.

[21] Kicha A.A., Ivanchina N.V., Kalinovsky A.I., Dmitrenok P.S., Stonik V.A. (2005). Structures of new polar steroids from the Far-Eastern starfish *Ctenodiscus crispatus*. Russ. Chem. Bull. Int. Ed..

[22] De Marino S., Iorizzi M., Zollo F., Minale L., Amsler C.D., Baker B.J., McClintock J.B. (1997). Isolation, Structure Elucidation, and Biological Activity of the Steroid Oligoglycosides and Polyhydroxysteroids from the Antarctic Starfish *Acodontaster conspicuus*. J. Nat. Prod..

[23] Minale L., Pizza C., Zollo F., Riccio R. (1983). Trace Polyhydroxylated steroids from starfish *Hacelia attenuata*. J. Nat. Prod..

[24] Finamore E., Minale L., Riccio R., Rinaldo G., Zollo F. (1991). Novel marine polyhydroxylated steroids from the starfish *Myxoderma platyacanthum*. J. Org. Chem..

[25] Wang W., Li F., Park Y., Hong J., Lee C.O., Kong J.Y., Shin S., Im K.S., Jung J.H. (2003). Bioactive sterols from the starfish *Certonardoa semiregularis*. J. Nat. Prod..

[26] Li Z., Chen G., Lu X., Wang H., Feng B., Pei Y. (2013). Three new steroid glycosides from the starfish *Asterina pectinifera*. Nat. Prod. Res..

[27] Djerassi C., Theobald N., Kokke W.C.M.C., Pak C.S., Carlson R.M.K. (1979). Recent progress in the marine sterol field. Pure Appl. Chem..

[28] Ferezou J.P., Devys M., Allais J.P., Barbier M. (1974). C26 sterols. IX. C26 sterol from the red alga *Rhodymenia Palmata*. Phytochemistry.

