# Supplementary material for: Phallusiasterol C, A New Disulfated Steroid from the Mediterranean Tunicate Phallusia fumigata
Source: Mar Drugs. 2016 Jun 18;14(6):117. doi: 10.3390/md14060117 (PMC4926076; doi:10.3390/md14060117)
Supplement: Supplementary file 1 [file marinedrugs-14-00117-s001.pdf]

Supplementary Material: Phallusiasterol C, A New Disulfated Steroid from the Mediterranean Tunicate *Phallusia fumigata*

Concetta Imperatore, Maria Senese, Anna Aiello, Paolo Luciano, Stefano Fiorucci, Claudio D'Amore, Adriana Carino and Marialuisa Menna

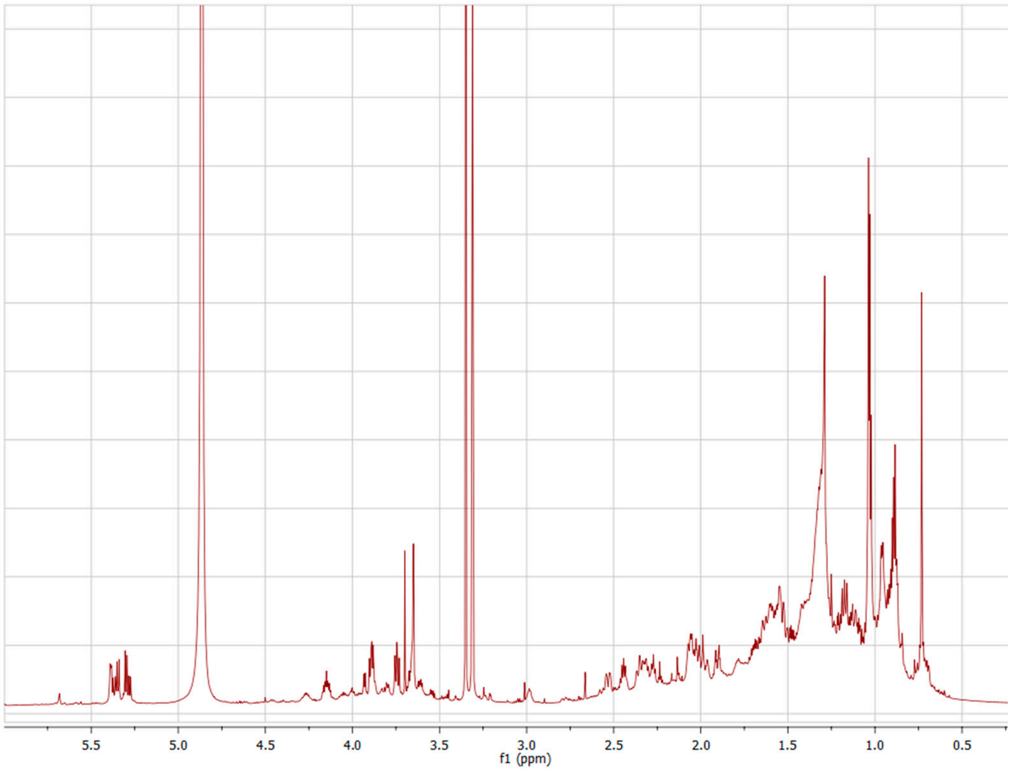

Figure S1. ^{1}H NMR spectrum of phallusiasterol C (**1**) (CD_3OD, 700 MHz).

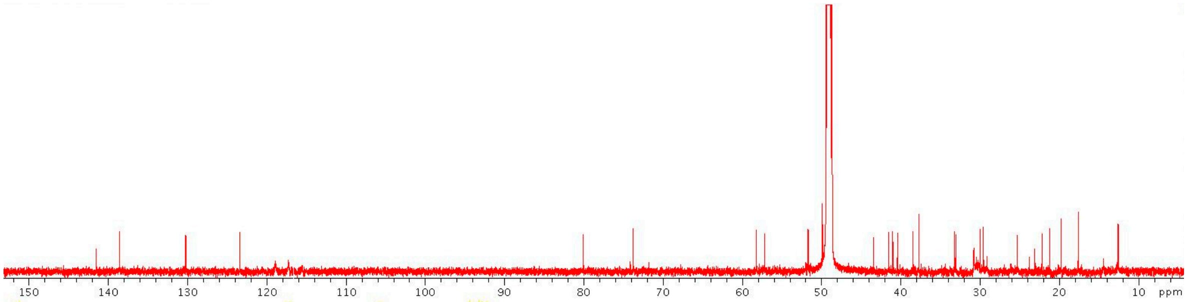

Figure S2. ^{13}C NMR spectrum of phallusiasterol C (**1**) (CD_3OD, 700 MHz).

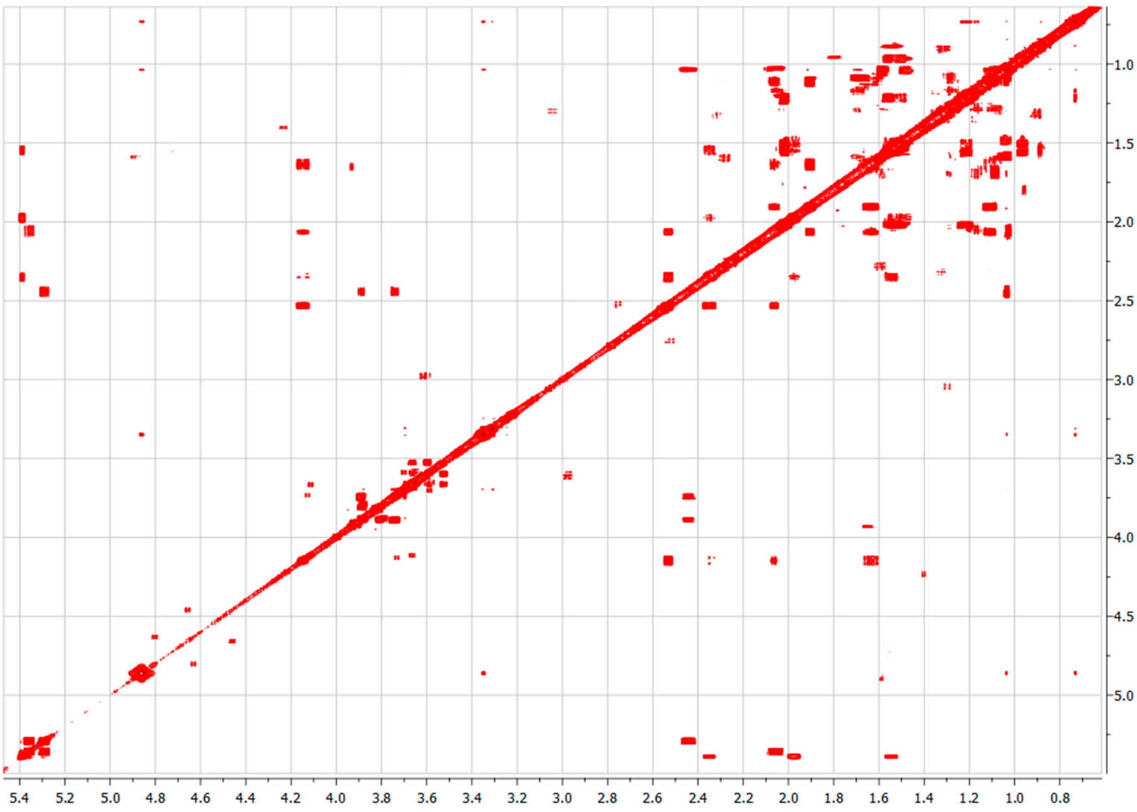

Figure S3. COSY spectrum of phallusiasterol C (**1**) (CD_3OD, 700 MHz).

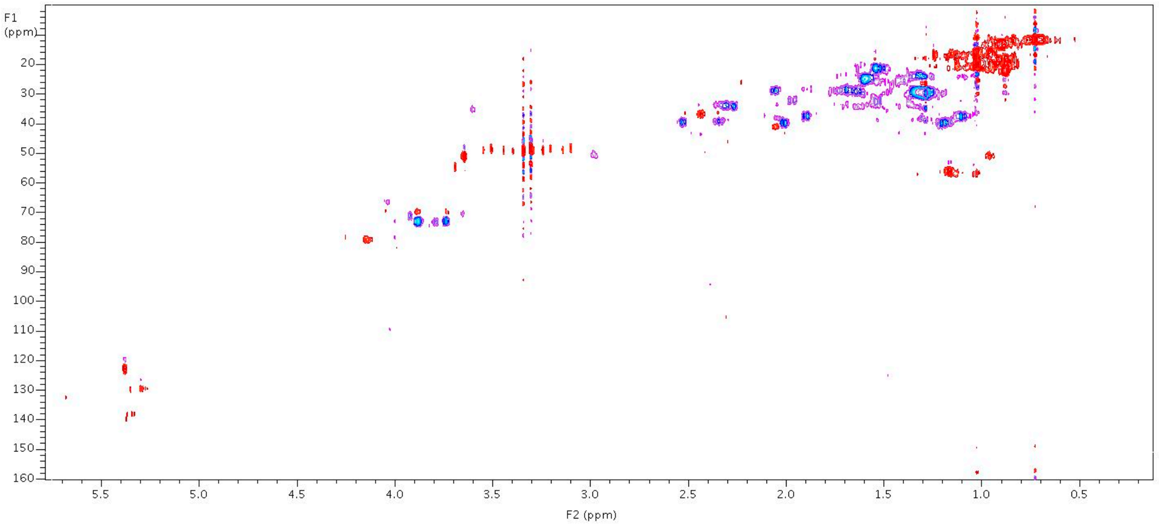

Figure S4. HSQC spectrum of phallusiasterol C (**1**) (CD_3OD, 700 MHz).

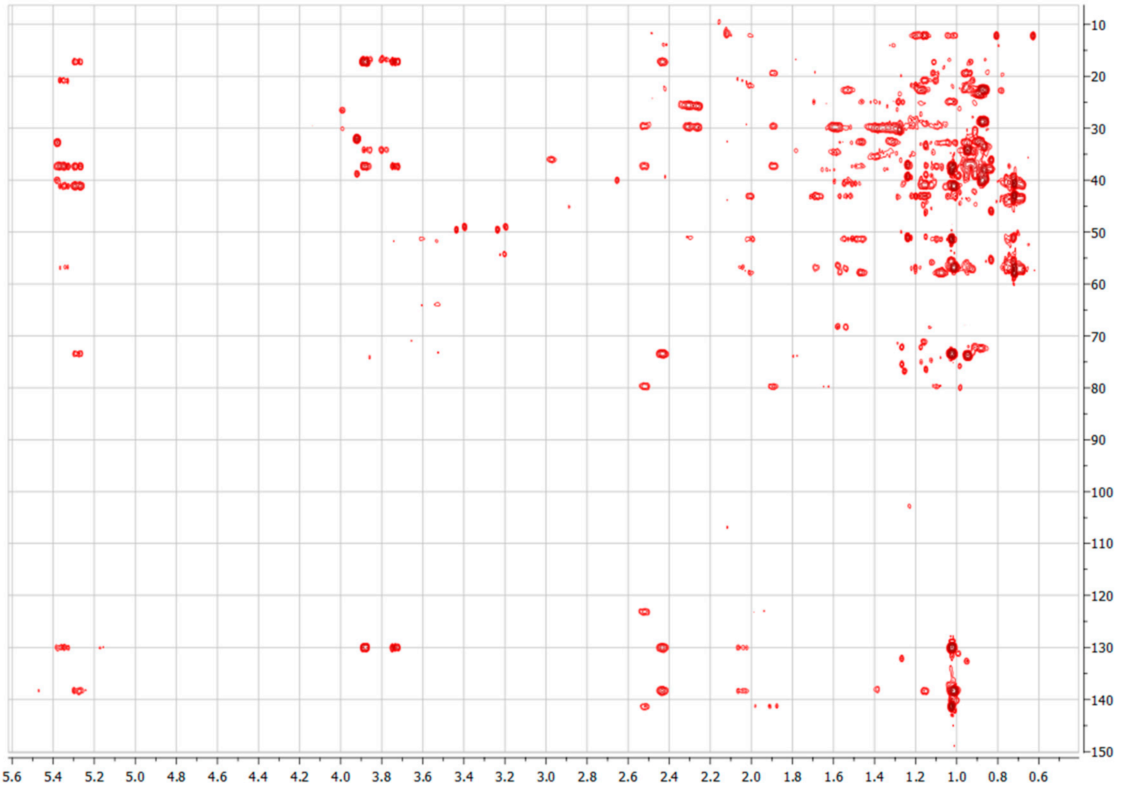

Figure S5. HMBC spectrum of phallusiasterol C (**1**) (CD_3OD, 700 MHz).

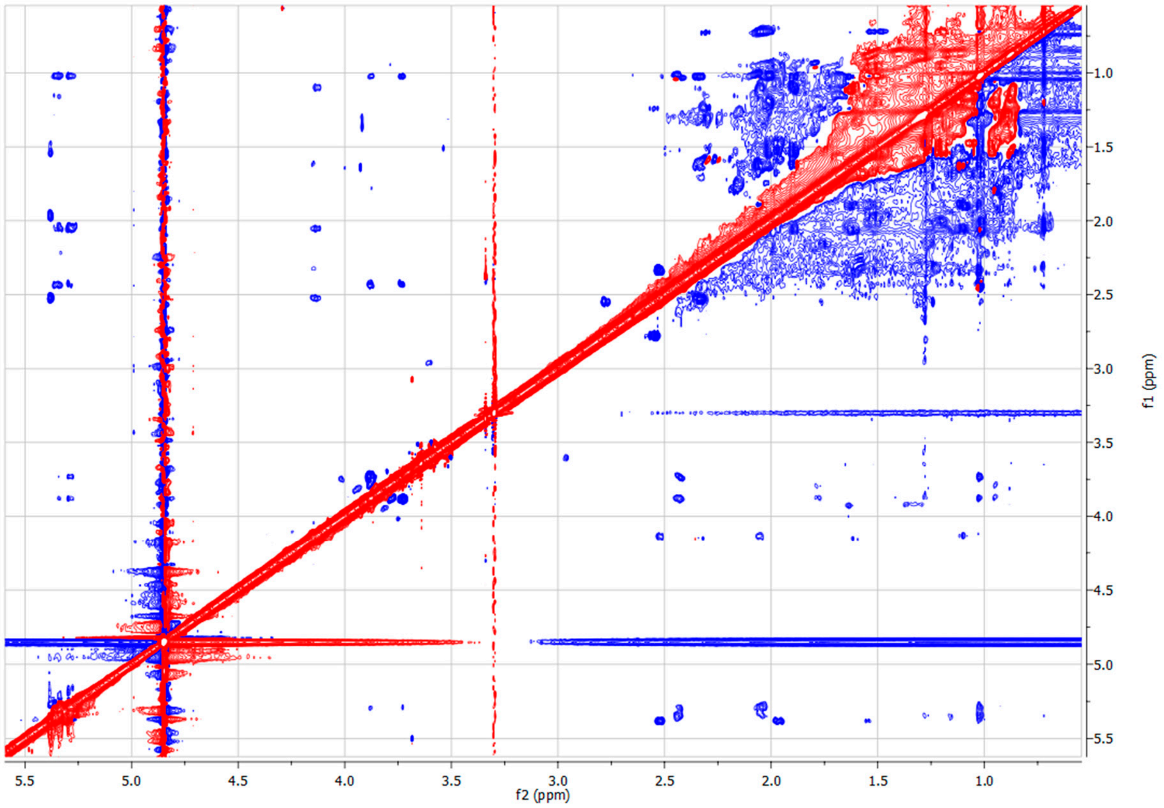

Figure S6. ROESY spectrum of phallusiasterol C (**1**) (CD_3OD, 700 MHz).

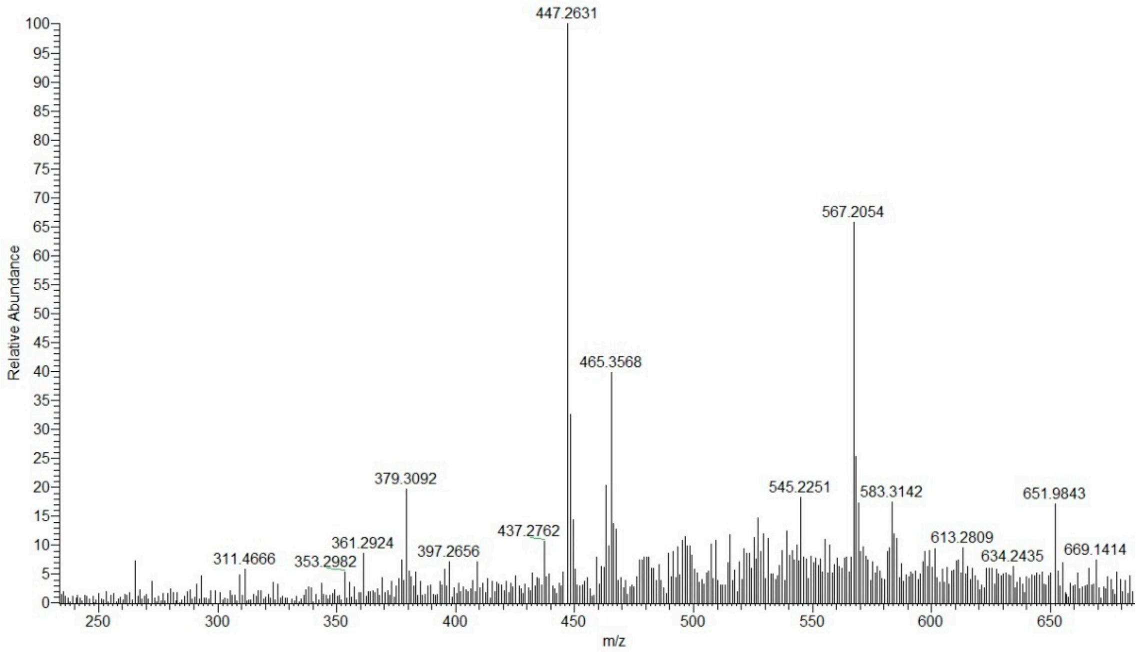

Figure S7. Negative-ion HRESI MS spectrum of phallusiasterol C (**1**).
